# Human cystic echinococcosis in Morocco: Ultrasound screening in the Mid Atlas through an Italian-Moroccan partnership

**DOI:** 10.1371/journal.pntd.0005384

**Published:** 2017-03-01

**Authors:** Houda Chebli, Abderrhamane Laamrani El Idrissi, Mustapha Benazzouz, Badre Eddine Lmimouni, Haddou Nhammi, Mourad Elabandouni, Mohammed Youbi, Rajaa Afifi, Sara Tahiri, Abdellah Essayd El Feydi, Adbellatif Settaf, Carmine Tinelli, Annalisa De Silvestri, Souad Bouhout, Bernadette Abela-Ridder, Simone Magnino, Enrico Brunetti, Carlo Filice, Francesca Tamarozzi

**Affiliations:** 1 Directorate of Epidemiology, Division of Infectious Diseases, Service of Parasitic Diseases, Ministry of Health of Morocco, Rabat, Morocco; 2 Department of Hepatogastroenterology (Medicine C), Ibn Sina Hospital, University “Mohammed V”, Rabat, Morocco; 3 Parasitology laboratory, Military Teaching Hospital “Mohammed V”, Rabat, Morocco; Team Research in parasitology, tropical and fungal infectious diseases, School of Medicine and Pharmacy, University “Mohammed V”, Rabat, Morocco; 4 Department of Hepatobiliary Surgery (Surgery B), Ibn Sina Hospital, University “Mohammed V”, Rabat, Morocco; 5 Clinical Epidemiology and Biometry Unit, San Matteo Hospital Foundation, Pavia, Italy; 6 Department of Control of Neglected Tropical Diseases, WHO Headquarters, Geneva, Switzerland; 7 Department of Clinical, Surgical, Diagnostic and Pediatric Sciences, University of Pavia, Pavia, Italy; WHO-Collaborating Centre for Clinical management of Cystic Echinococcosis, Pavia, Italy; 8 Division of Infectious and Tropical Diseases, San Matteo Hospital Foundation, Pavia, Italy; University of Zurich, SWITZERLAND

## Abstract

**Background:**

Cystic echinococcosis (CE) is a neglected parasitic zoonosis with considerable socioeconomic impact on affected pastoral communities. CE is endemic throughout the Mediterranean, including Morocco, where the Mid Atlas is the most prevalent area for both human and animal infection. The highest hospital annual incidence of human CE is recorded in the provinces of Ifrane and El Hajeb. However, hospital-based statistics likely underestimate the real prevalence of infection, as a proportion of cases never reach medical attention or official records.

**Methodology/Principal findings:**

In 2012, a project on clinical management of CE in Morocco was launched with the aims of estimating the prevalence of human abdominal CE in selected rural communes of the above mentioned provinces using ultrasound (US) screening and training local physicians to implement US-based focused assessment and rational clinical management of CE according to the WHO-IWGE Expert Consensus. A total of 5367 people received abdominal US during four campaigns in April-May 2014. During the campaigns, 24 local general practitioners received >24 hours of hands-on training and 143 health education sessions were organized for local communities. We found an overall CE prevalence of 1.9%, with significantly higher values in the rural communes of Ifrane than El Hajeb (2.6% vs 1.3%; p<0.001). CE cysts were predominantly in inactive stage, especially in older age groups. However, active cysts were present also in adults, indicating acquisition of infection at all ages. Province of residence was the only risk factor consistently associated with CE infection.

**Conclusions/Significance:**

Our results show a high prevalence and on-going, likely environmental transmission of CE in the investigated provinces of Morocco, supporting the implementation of control activities in the area by national health authorities and encouraging the acceptance and divulgation of diagnosis and treatment algorithms based on imaging for CE at both national and local level.

## Author summary

Cystic Echinococcosis (CE) is a parasitic infection whose natural domestic cycle develops between dogs and sheep (and other livestock). Human infection is endemic in pastoral communities, where close contact with the dog-sheep cycle occurs. In humans, as well as in livestock, the parasite develops as fluid-filled cyst mainly in the liver. CE is a neglected disease, as it is a disabler without high mortality, it affects mainly poor communities, and requires complex and expensive clinical management and long-term integrated public health control strategies. The prevalence of infection in an area is often unknown or largely underestimated, therefore the problem is perceived as not important. In Morocco, the Mid Atlas is the most prevalent area for human and animal infection. We performed an ultrasound survey on 5,367 people in Ifrane and El Hajeb provinces, and found an overall prevalence of 1.9%. CE cysts were predominantly inactive, however, active cysts were present also in adults. Our results show a high prevalence and on-going transmission of CE, encouraging the prompt strengthening and complete implementation of control activities envisaged by Moroccan health authorities in the area and the adoption of diagnosis and treatment algorithms based on imaging at both national and local level, to avoid a risk-associated and expensive treatment of inactive cysts.

## Introduction

Cystic Echinococcosis (CE) is a globally distributed parasitic zoonosis, caused by the larval stage of the dog tapeworm *Echinococcus granulosus* sensu lato. Its life cycle develops between the dog (and other canids), which is the definitive host harbouring the adult parasites in the intestine, and the sheep (and other ungulates), which is the intermediate host where the larval form may develop in organs and tissues. Humans, which are aberrant “dead-end” intermediate hosts, as well as natural intermediate hosts, become infected through ingestion of eggs released with the faeces of parasitized dogs. Eggs can remain viable for months in the environment. In the intermediate host, the parasite larvae develop in organs and tissues, mainly the liver and the lungs, forming fluid-filled cysts (commonly referred to as hydatid cysts) that expand centrifugally. The cycle completes when the definitive host ingests viscera infected with hydatid cysts.

CE affects mainly pastoral communities where close contact with the dog-sheep cycle and a contaminated environment occur. However, clear-cut association with risk factors is still lacking [1]. The global burden of CE has been estimated in 1.2 million people infected worldwide with over 3.5 million DALYs (Disability-Adjusted Life Years) lost globally every year [2]. However, these figures are likely underestimated. The geographical dispersal of the infection on vast rural areas with a patchy distribution, the absence of specific symptoms, and the lack of an effective disease record system, hamper a more precise assessment of infection prevalence and disease burden. Furthermore, CE mostly affects poor communities, is a disease with a low case-fatality rate with difficult and expensive diagnosis and treatment, and is an infection with a transmission cycle that is difficult to interrupt in the absence of sustained, expensive and well-coordinated programs involving both human and veterinary health services [2]. As a result, this disease is neglected [2, 3].

Morocco is highly endemic for CE. The prevalence of infection in animals has been reported to be up to 58.8% in dogs, 19.3% in sheep and 48.7% in cattle, with consistent variations between regions (Guide des activites de lute contre l’hydatidose/echinococcose. Comité Interministeriel de lute contre l’hydatidose/echinococcose, Royaume du Maroc, 2007). The Mid Atlas region was found to be the most prevalent area for animal CE in surveys conducted between 2001 and 2004 by Azlaf and Dakkak [4].

A total of 23,512 operated human cases were recorded by the Ministry of Health of Morocco during the periods 1980–1992 and 2003–2008 [5]. An increasing average annual incidence of surgical cases was shown, from 3.6 to 5.2 per 100,000 inhabitants in 1980 and 2008, respectively, with the region of Meknes-Tafilalet, in the Mid Atlas, recording the highest figures (11.9 per 100,000 in 2008) [5]. More recent data from the Ministry of Health of Morocco for 2014 report an annual incidence of human CE in the Meknes-Tafilalet region of 7.04 per 100,000, with the highest figures recorded in the provinces of Ifrane (16.33 per 100,000) and El Hajeb (12.90 per 100,000). These figures, however, are likely not representative of the real prevalence of infection, as a proportion of cases remain clinically silent often for many years and, even when symptomatic, may never reach medical attention or official disease records. More accurate data may come from screening campaigns, which allow to detect also asymptomatic cases and to evaluate the distribution of CE stages among age groups. In 2000 and 2001, Macpherson and colleagues conducted an ultrasound (US) screening of 11,612 people in the provinces of Ifrane and Khenifra in the Meknes-Tafilalet region, finding a prevalence of abdominal CE of 1.1% (1.3% in Ifrane and 0.9% in Khenifra) [6]. Most of the diagnosed CE cases were asymptomatic, even in patients with large active cysts, and symptoms, when present, were non-specific.

The diagnosis and clinical management of CE are complex and require a multidisciplinary approach, often available only in referral centres. In 2003, the World Health Organization Informal Working Group on Echinococcosis (WHO-IWGE) implemented a consensus US-based classification of CE cysts stages, which allows classifying unequivocally all morphological stages of cysts [7]. This classification also groups cyst stages into clinical categories to guide the rational stage-specific allocation of CE patients to different management options, including surgery, medical therapy, percutaneous treatment and the “watch and wait” approach [8, 9]. This approach not only allows choosing the most appropriate treatment (or the need for no treatment) depending on cyst characteristics, patient-related factors, and therapeutic resources available, but also helps in rationalizing the expenses for CE management. Unfortunately, the use of this consensus approach, and of CE classifications of any kind, are still appallingly scarce, and the management of the disease is often inappropriate, exposing patients (and health systems) to unnecessary treatments, risks, and costs [10, 11]. A survey on clinical practices in CE carried out in Morocco in 2008 found that 73.6% of interviewed physicians (n = 148) would treat inactive CE cysts with surgery, only 4.1% would use the (correct) watch and wait approach, while 20.3% of the interviewed did not know what approach to take with inactive cysts [12]. In Morocco, the direct cost of surgery for abdominal CE has been estimated at 1500–3000 US$ per patient [4, 5]. Further costs include the reduction or loss of income due to hospitalization and complications and, not less important, the impact on patient’s quality of life due to the risks, complications, and long hospital stay associated with surgery. Nonetheless, the treatment of CE in Morocco is still almost uniquely surgical, while other options such as percutaneous treatment are implemented in only a minority of cases [5]. According to data from the Ministry of Health of Morocco, in 2014, percutaneous treatment was used in 1.8%, and medical treatment in 0.9% of recorded CE cases.

In 2004, an inter-ministerial committee for the control of CE was established in Morocco, involving the Ministry of Agriculture, the Ministry of Health and the Ministry of Interior. The committee started its activities in 2009. However, the implementation of integrated control measures proved extremely difficult due to problems of inter-ministerial collaboration at the organizational and funding level, and so far only improvement of human CE case report system has been implemented. A re-evaluation of the prevalence of human CE was therefore considered necessary.

In 2012, an EUR 300,000 project “Clinical Management of Cystic Echinococcosis in Morocco” funded by the Italian Ministry of Health and coordinated by the WHO was launched, with the aims of: i) estimating the prevalence of human abdominal CE in target endemic areas of Morocco using US screening; ii) encouraging the use of US-based focused assessment of CE in peripheral endemic areas [13, 14]; and iii) encouraging a rational clinical management of CE by local physicians according to the WHO-IWGE Expert Consensus on diagnosis and management of human echinococcosis [8]. Here we present the results of the community-based US screening and related activities carried out between April and May 2014 in the target endemic provinces of Ifrane and El Hajeb, Meknes-Tafilalet region (designated as such until December 2014 and indicated as such in the manuscript), Mid Atlas, an area among the most endemic in Morocco for both human and animal infection (Guide des activites de lute contre l’hydatidose/echinococcose. Comité Interministeriel de lute contre l’hydatidose/echinococcose, Royaume du Maroc, 2007; and [4, 5]).

## Materials and methods

### Ethics statement

Approval was granted by the Ethics Committees of the University of Pavia, Italy, and of the University Hospital Centre Hassan II of Fès, Morocco.

### Participating institutions

The project developed as a 2-year collaboration between the University of Pavia, San Matteo Hospital Foundation, WHO Collaborating Centre for the Clinical Management of Cystic Echinococcosis, Pavia, Italy, and the Ministry of Health of Morocco, Service of Parasitic Diseases, Rabat, Morocco. The project was coordinated by the WHO, Department of Neglected Infectious Diseases and the WHO Regional Office for the Eastern Mediterranean, Cairo, Egypt. Moroccan study centres were represented by the Service of Medicine C and the Service of Surgery B, Avicenne (Ibn Sina) hospital, Rabat; the Service of Parasitology, Mohammed V Military Teaching Hospital and Faculty of Medicine and Pharmacy, Rabat; the Prince Moulay Hassan Hospital, El Hajeb; and the 20 August Hospital, Ifrane. Local support and coordination was provided by the WHO Office in Morocco, Rabat, the Regional Health Directorate of Meknes-Tafilalet region, and the Provincial Delegations of Ifrane and El Hajeb.

### Objectives

The main objective of this cross-sectional study was to estimate the prevalence of abdominal CE in the target endemic provinces of Ifrane and El Hajeb, Meknes-Tafilalet region, Mid Atlas. Secondary objectives were i) to assess risk factors associated with CE infection, ii) to train local health professionals on the diagnosis and clinical management of abdominal CE according to the WHO-IWGE Expert Consensus on diagnosis and management of human echinococcosis [8] and, iii) to provide educational inputs on the disease, its transmission, treatment and prevention, to the inhabitants of the communities involved in the screening. The evaluation of the effectiveness of the stage-specific clinical management of patients with abdominal CE according to the WHO-IWGE Expert Consensus is on going and will be the object of a further publication.

### Sample size calculation and data analysis

A sample size of 5,000 subjects (2,500 per province) was calculated to provide an estimate of CE prevalence with a 95% confidence level and 0.5% precision, based on an expected prevalence of 1.5%. The Shapiro-Wilk test was used to assess the normal distribution of quantitative variables. These were expressed as the mean and standard deviation, as they were normally distributed, and were compared by t-test. Qualitative variables were described as number and percentage. Differences between groups were evaluated using χ2 or Fisher’s exact test, as appropriate. Association between CE infection and potential risk factors, with the exception of age and sex, were assessed using univariate logistic regression models. To take into account potential confounders we calculated Odds Ratios (ORs, with their 95% Confidence Interval) adjusted for age, sex, and province by multivariable logistic models. Each type of “dog role” was analyzed as “yes/no” as dogs may have several roles in the same households. At cyst level, risk of being in active or inactive stage was assessed by logistic regression with robust standard errors, clustered at patient level. As missing data were below 5%, no statistical method for missing data was necessary. A p-value < 0.05 was considered statistically significant and a p-value < 0.10 was considered borderline significant. All tests were two sided. Data analysis was performed with the STATA statistical package (version 14.1; Stata Corporation, College Station, TX, USA).

### Ultrasound survey

**Fig 1:**
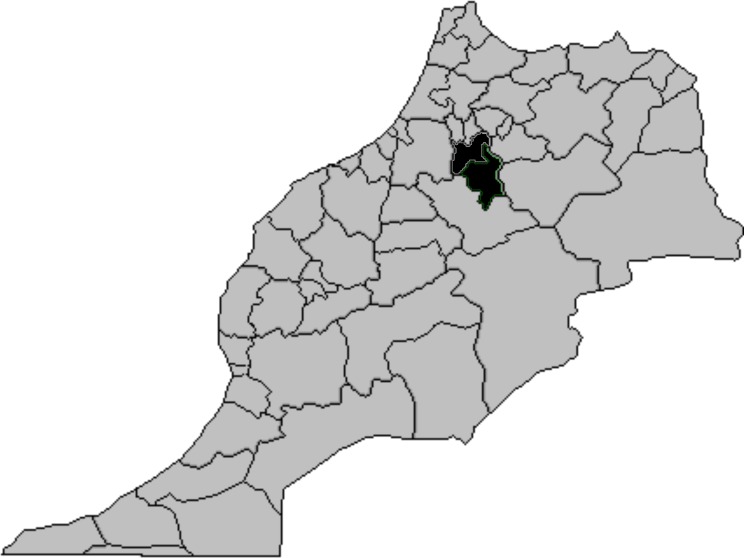
Map of Morocco and the survey area. The survey area is indicated in black. This includes the rural communes of Timahdit and Ain Louh in the province of Ifrane, and the rural communes of Bouderbala and Sebt Jehjouh in the province of El Hajeb, Meknes-Tafilalet region (designated as such until December 2014). Modified from Di Hanhil [Public Domani] Wikimedia Commons.

Consent to conduct the screening was obtained from local authorities and community leaders during the preparatory phase of the project. The surveys comprised four 2-day campaigns in April and May 2014, in the rural communes of Ain Louh and Timahdit in the province of Ifrane, and in the rural communes of Bouderbala and Sebt Jehjouh in the province of El Hajeb, Meknes-Tafilalet region (Fig 1). The main population of these areas of central Morocco are Amazighs. The rural communes of Ain Louh and Timahdit in Ifrane province are located at an altitude of 1300 and 1900 m a.s.l. and are 24 km and 34 km distant from the closest city, respectively. Their population is 9669 and 10945 inhabitants, respectively. The rural communes of Bouderbala and Sebt Jehjouh in the province of El Hajeb are located at a lower altitude, between 760 and 1000 m a.s.l. They are located 16 km and 15 km, respectively, from the closest city, with a population of 7907 and 7485 inhabitants, respectively (data from the Haut Commissariat au Plan, Morocco, 2014). These areas were chosen after evaluation of available human and animal data on the presence of CE, and agreement from local authorities. According to official data of the Ministry of Agriculture and Fishery of Morocco, in 2014 the province of Ifrane counted 900,000 ovines, with a prevalence of CE infection in this species of 12.2%, while the province of El Hajeb counted 330,000 ovines, with a prevalence of CE infection of 3.5%. These figures are considerably lower than those reported by the Comité Interministeriel de lute contre l’hydatidose/echinococcose (2007), an underestimation possibly in part deriving from the fact that abattoirs survey data reflect the age of the animals slaughtered. Indeed, young animals are less infected than older ones, and, in addition, data from abattoirs are cumulative, not differentiating between age categories, therefore inducing bias and underestimation.

On average 40 staff members were involved in the implementation of each campaign. Pre-screening activities included two preliminary meetings with all staff involved, inspections to the screening sites, and regular announcement of the screening dates to the population during social and religious occasions (weekly markets, Mosques, etc). The screening activities were carried out in primary school buildings, in each of which eight rooms were used: one for the registration and waiting of participants; one for the health education sessions, one for the administration of the Participant Information Sheet and the risk factors questionnaire, and the signature of the Informed Consent Form; four for clinical examination using eight portable ultrasound machines, and one for blood sampling, medical interview and counselling of patients diagnosed with CE. All residents of the target study areas aged between 10 and 80 years were invited to take part into the screening. However, younger children brought by their parents and older persons responding to the invitation were also examined. The Informed Consent Form was signed by the parent/legal representative for subjects <18 years of age. Males and females were examined by US in separate rooms.

### Risk factors questionnaire

All participants were asked to answer a risk factors questionnaire before US examination. The questionnaire was written in French and verbally translated into the local language Amazigh by the survey staff. Questions included recognition of CE cysts upon show of a picture of an infected sheep liver, information on main water source for human use, livestock breeding, home slaughter, disposal of offal, ownership and management of owned dogs (purpose of dog ownership, dog confinement inside and outside the owner’s premises, feeding habits, deworming with praziquantel, use of same source of water by dogs and livestock), and access of unowned dogs to the premises

### Patient classification

All patients diagnosed with abdominal CE or suspected CE lesions/surgical scars were asked about previous CE diagnosis and treatment, underwent blood sampling for the examination of laboratory parameters and CE serology, and received a chest X ray for the detection of possible pulmonary CE lesions. All women of childbearing age were also assessed for pregnancy using hCG urine rapid test. After receiving detailed information and counselling about the most appropriate clinical management according to the WHO-IWGE Expert Consensus indications, patients were invited to sign the Informed Consent Form to treatment. Subjects <18 years of age were addressed to a paediatric hospital for treatment. Treatment was offered free of charge.

Patients were classified as positive for abdominal CE if: i) they had abdominal lesions with pathognomonic features of CE at US irrespective of their serology results; or if ii) they had abdominal lesions compatible with CE and positive serology; or if iii) they had post-treatment lesions from previous CE treatment. In the latter case, where a residual cavity was visualized on US with suspect features for relapse, a diagnostic puncture was proposed and the cyst fluid analysed microscopically and by PCR (see below) to define the nature of the lesion.

Suspect lesions were investigated with diagnostic puncture, Magnetic Resonance Imaging, and US re-evaluation, as appropriate. All subjects diagnosed with medically relevant conditions other than CE were referred to the reference provincial hospital or regional hospital, as appropriate, for free of charge treatment according to the agreement with the Ministry of Health of Morocco.

### Serology and cyst fluid genotyping

Serology for echinococcosis was performed using ELISA (RIDASCREEN Echinococcus IgG, R-Biopharm, Darmstadt, Germany) and Western Blot (WB) (Echinococcus IgG, LD-BIO Diagnostics, Lyon, France) according to manufacturers’ instructions. For the analysis, patients with both hepatic and extra-hepatic cysts were classified into 3 groups according to the stage of the hepatic cyst. When more than one hepatic cyst was present, patients were grouped according to the stage of the cyst known to have the most influence on a positive serology result, i.e. CE2-CE3a-CE3b>CE1>CE4-CE5 [15]. To assess the *E*. *granulosus* genotypes infecting CE patients, hydatid fluids available after percutaneous or surgical interventions of patients with CE or suspected CE were analysed by PCR according to the method described by Boubaker et al [16].

### Training and educational activities

Before the survey, a 1-day meeting was organized, directed to the medical personnel of the target areas involved in the study. The workshop included 6 hours of frontal lectures on the current techniques of diagnosis and recommendations on the clinical management of CE. Educational material was also provided to the participants. Twenty-four general practitioners practicing in the two target provinces (12 per province) received >18 hours of hands-on training on general abdominal US and focused assessment of CE with US during the screening campaigns, by flanking expert sonographers conducting the screening. None of the physicians had ever received training on CE and ultrasonography before the project. Physicians from El Hajeb specialized in gastroenterology and surgery (one per specialty) agreed to receive practical training on surgical and percutaneous CE treatment techniques at the Avicenne (Ibn Sina) hospital, Rabat, during the treatment of diagnosed patients. Unfortunately physicians from Ifrane were not available for training during the envisaged period. Educational posters and handouts in Arabic were made available in the places of the US screening to inform the local population on the infection, its transmission, and its prevention. Participants were organized in groups of 30–35 people and received a 15-minute presentation by the Provincial Training Facilitator on Information, Education, and Communication with the aid of simple Power Point slides written in Arabic and explained in the local language Amazigh. Educational material was also provided to the participants.

## Results

### Demographic characteristics of the screened population

**Fig 2:**
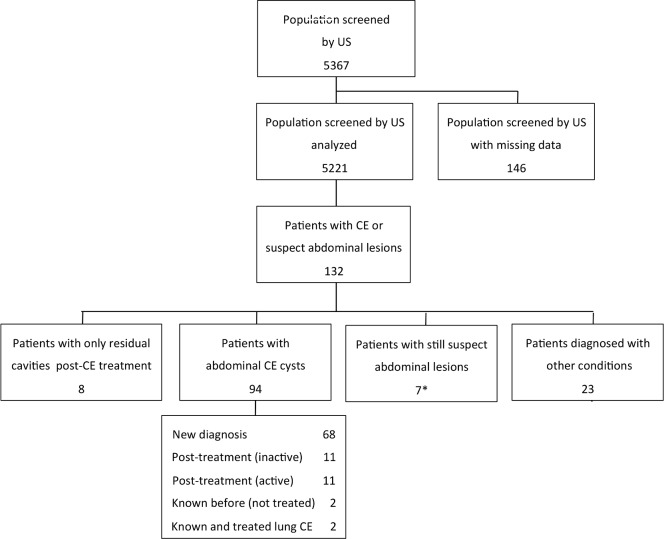
Examined people and CE cases observed during the screening campaigns. *One patient reported previous surgery for pulmonary CE.

A total of 5,367 people, aged 3–94 years, participated voluntarily in the screening and were evaluated by abdominal US during the 4 campaigns, of which 2,705 (50.4%) in Ifrane and 2,662 (49.6%) in El Hajeb. This constituted 1.7% of the whole population of Ifrane in 2014 and 13.1% of that of the two investigated rural communes in this province; and 1.1% of the whole population of El Hajeb and 17.3% of the two investigated rural communes in this province (data from the Haut Commissariat au Plan, Morocco, 2014). During the screening campaigns, 143 health education sessions were performed, for a total of 5249 local people. Of the 5367 people screened, data were available for analysis from 5221 people (97.3%), with results seen in Fig 2, of which 2633 from Ifrane and 2588 from El Hajeb. The majority (70.7%) of screened people were females. The demographic and social distribution of the general resident population and the screened population is detailed in Table 1.

**Table 1:** Demographic characteristics distribution of the screened and general population of the investigated provinces. Three children (aged 3, 8 and 9 years) and 19 adults (aged 81–94 years) were outside the target age range.

	Screened population Ifrane	Screened population El Hajeb	General population Ifrane	General population El Hajeb
**Gender**				
**Male**	840 (31.9%)	691 (26.7%)	48.9%	49.8%
**Female**	1793 (68.1%)	1897 (73.3%)	51.1%	50.2%
**Age (years)**				
**Mean (SD)**	40.4 (18.0)	39.7 (17.6)		
**3–20**	448 (17.0%)	504 (19.5%)	39.6%	37.1%
**21–40**	972 (36.9%)	931 (35.8%)	33.1%	33.5%
**41–60**	803 (30.5%)	866 (33.5%)	18.2%	17.5%
**61–80**	398 (15.1%)	268 (10.4%)	(>60 years) 9.1%	(>60 years) 7.8%
**81–94**	4 (0.2%)	15 (0.6%)		
**Missing data**	8 (0.3%)	4 (0.2%)		
**Occupation**				
**Livestock raising/ Agriculture**	395 (15.0%)	212 (8.2%)		
**Student**	348 (13.2%)	343 (13.3%)		
**Labourer**	154 (5.9%)	141 (5.4%)		
**Housewife**	1394 (52.9%)	1326 (51.2%)		
**Other**	99 (3.8%)	86 (3.3%)		
**Unemployed**	132 (5.0%)	92 (3.6%)		
**Missing data**	111 (4.2%)	388 (15.0%)		

### Prevalence of CE and risk factors analysis

The number of examined people and CE cases found during the screening campaigns are summarized in Fig 2. Of the 5,221 people who received abdominal US, 132 subjects had at least one abdominal CE or suspect abdominal lesion. Of these, 102 subjects (1.9% [95% CI 1.6%-2.4%]) had at least one abdominal CE lesion, either a CE cyst (92.2%), or a residual lesion from previous surgery for abdominal CE (7.8%). CE was excluded in 32 subjects while for 7 people the aetiology of the lesion is still not determined at the time of writing. As shown in Table 2, the prevalence in the province of Ifrane (2.6% [95% CI 2.0%-3.3%]) was significantly higher than that of El Hajeb (1.3% [95% CI 0.9%-1.8%]) (p<0.001). Using logistic regression analysis, the risk of having CE was significantly higher in the two investigated rural communes of Ifrane province (OR 2.8 [95% CI 1.3–5.7] in Ain Louh and OR 3.3 [95% CI 1.6–6.7] in Timahdit) compared to the rural commune of Sebt Jahjouh in the province of El Hajeb (p = 0.005 and 0.001, respectively), showing the lowest prevalence, while there was no significant increased risk compared to this area in the second investigated rural commune of Bouderbala in the El Hajeb province (OR 1.8 [95% CI 0.9–3.9]; p = 0.114).

**Table 2:** Population screened and prevalence of abdominal CE in the investigated provinces and rural communes.

Province	Rural commune	N screened	CE cases	Prevalence (95% CI)	p-value between provinces	OR (95% CI) compared with Sebt Jahjouh	p-value compared with Sebt Jahjouh
Ifrane	Timahdit	1336	38	2.8% (2.0%-3.9%)	**p<0.001**	3.3 (1.6–6.7)	**p = 0.001**
	Ain Louh	1297	31	2.4% (1.6%-3.4%)		2.8 (1.4–5.7)	**p = 0.005**
	Total	2633	69	2.6% (2.0%-3.3%)			
El Hajeb	Bouderbala	1447	23	1.6% (1.0%-2.4%)		1.8 (0.9–3.9)	p = 0.114
	Sebt Jahjouh	1141	10	0.9% (0.4%-1.6%)		-	-
	Total	2588	33	1.3% (0.9%-1.8%)			
Total		5221	102	1.9% (1.6%-2.4%)			

The association between CE infection and each risk factor (Table 3) was investigated taking into account province and age and gender, which were not significantly different between CE positive and CE negative subjects (age p = 0.734 and p = 0.856, and gender p = 0.896 and p = 0.166 in Ifrane and El Hajeb, respectively). Province of residence was constantly found associated with high statistical significance with CE infection (p<0.001 in univariate logistic regression model; p<0.01 in all multivariable logistic models). Dog ownership was found to be associated with borderline statistical significance (p = 0.063) with CE infection only in Ifrane province, where the proportion of infected subjects owning a dog was 68% vs 57% of non infected subjects, and statistically associated (p = 0.035) with infection when adjusting for age, sex, and province. Other variables associated with borderline significance to infection were livestock breeding in the household (p = 0.098), owned dogs allowed to roam (p = 0.099, only for Ifrane province), and raw viscera given to dogs (p = 0.077, only in El Hajeb province). The proportion of people recognizing CE lesions in pictures of animal infected organs was very high (93.1% in Ifrane and 57.8% in El Hajeb). Although the questionnaire was administered after the health education session, and therefore answers may have been influenced by the recently heard information, it is possible they were true recognition of the parasitic lesion, as the absence of abattoirs in these rural communes makes inhabitants exposed to infected organs after home slaughter.

**Table 3:** Risk factor analysis for CE infection.

	Crude OR (95%CI)		Adjusted OR ** (95%CI)	P value°
	Ifrane	El Hajeb		
Age (per year)	1.0 (0.9–1.1)	1.0 (0.9–1.1)		
Sex (M vs F)	0.9 (0.5–1.5)	0.5 (0.2–1.3)		
Recognition of *E*. *granulosus* cysts (yes/no)	0.9 (0.4–1.4)	1.1 (0.5–2.2)	0.9 (0.6–1.8);	p = 0.970
Main type of water source				
Open source ^	1	1	1	
Tap	1.2 (0.7–2.3)	1.0 (0.2–4.2)	1.2 (0.7–2.2)	p = 0.440
Public fountain	1.1 (0.6–1.9)	1.7 (0.8–3.5)	1.3 (0.8–2.1)	p = 0.208
Livestock breeding in the household (yes/no)	1.2 (0.7–1.9)	1.8 (0.9–3.6)	1.4 (0.9–2.1)	p = 0.098*
Home livestock slaughtering (yes/no)	1.2 (0.5–2.9)	1.3 (0.6–2.9)	1.2 (0.7–2.3)	p = 0.451
Main way of viscera disposal				
Given to dogs^	1	1	1	
Buried	1.2 (0.5–2.8)	0.9 (0.1–4.2)	1.1 (0.5–2.2)	p = 0.870
Used as fertilizer	2.4 (0.6–10)	2.0 (0.4–11)	2.3 (0.5–9.8)	p = 0.273
Burned	1.1 (0.7–2.3)	0.9 (0.8–2.1)	1 (0.8–2.0)	p = 0.900
Thrown outside/garbage	1.9 (0.7–4.9)	0.5 (0.1–2.2)	1.1 (0.5–2.4)	p = 0.833
Dog ownership (yes/no)	1.6 (1.0–2.7)	1.3 (0.6–2.6)	1.6 (1.1–2.4)	**p = 0.035**
Role of owned dogs				
Guard (yes/no)	1.4 (0.2–11)	1.3 (0.2–10)	1.4 (0.3–5.8)	p = 0.653
Shepherd (yes/no)	1.2 (0.6–2.4)	0.6 (0.1–2.3)	0.9 (0.5–1.8)	p = 0.999
Pet (yes/no)	0.8 (0.4–1.9)	1.6 (0.2–12)	0.9 (0.4–1.9)	p = 0.781
Hunting (yes/no)	0.8 (0.6–2.9)	1.2 (0.7–3.9)	1 (0.8–1.9)	p = 0.910
Owned dogs allowed to roam (yes/no)	2.8 (0.9–9.0)	1.2 (0.4–3.2)	1.8 (0.9–3.7)	p = 0.115
Owned dogs allowed to enter the house (yes/no)	1.4 (0.7–2.5)	1.3 (0.4–3.8)	1.4 (0.8–2.4)	p = 0.227
Raw viscera given to dogs (yes/no)	1.7 (0.7–3.9)	0.4 (0.2–1.1)	1.0 (0.6–1.9)	p = 0.894
Owned dogs dewormed with praziquantel (yes/no)	1.4 (0.8–2.8)	1.6 (0.8–2.8)	1.5 (0.8–2.7)	p = 0.197
Unowned dogs can enter the premises (yes/no)	1.4 (0.8–2.4)	0.8 (0.4–1.9)	1.2 (0.8–1.9)	p = 0.390
Dogs and livestock use same water source (yes/no)	0.9 (0.3–3.1)	1.2 (0.4–2.9)	1.1 (0.4–2.8)	p = 0.988

Association between CE infection and each variable; Odds Ratio (ORs) adjusted for age, sex and province are shown

*Borderline significant

**OR (95%CI) adjusted for age, sex and province

° P-value for adjusted OR

^Reference value.

### CE cysts characteristics

The characteristics of CE cases found during the screening campaigns are summarized in Fig 2. Of the 102 subjects classified as having abdominal CE, 94 (92.2%) had CE cyst in abdominal organs, while 8 (7.8%) patients had only residual lesions from previous surgery for abdominal CE. None of the patients with abdominal CE had lung infection, as assessed by X ray of the chest. Twenty-three patients with suspect CE lesions were excluded from having the parasitic infection after re-examination, as appropriate. The alternative diagnoses were biliary cysts (n = 15), haemangioma (n = 1), hepatocellular carcinoma (n = 1), other kidney diseases (n = 4), and absence of lesions at re-evaluation (n = 2).

**Fig 3:**
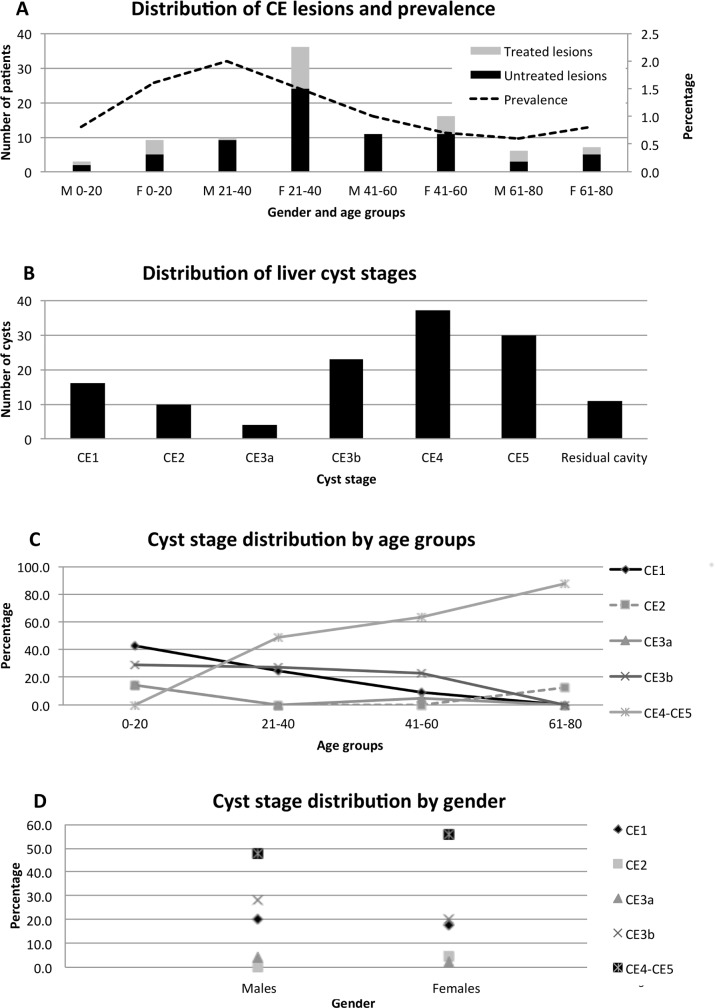
Distribution of CE lesions. **A.** Number of subjects with untreated and previously treated CE cysts (bars; left Y axis) and overall prevalence of CE in the screened population (dotted line; right Y axis) according to gender and age group (X axis). M = males; F = females. Age groups are expressed in years. Three children (aged 3, 8 and 9 years) and 19 adults (aged 81–94 years) were outside the target age range target; none of these subjects were infected with CE. **B.** Number of hepatic CE cysts in different stages. **C.** Percentage of CE cyst stages in patients never previously treated for CE within age groups. **D.** Percentage of CE cyst stages in patients never previously treated for CE within gender groups.

Of the 94 patients with abdominal CE cysts, 68 (72.3%) did not know they were infected, and were therefore newly diagnosed. The remaining 26 (27.7%) patients already knew about their condition. Infected patients were symptomatic in 47.9% of cases; the most frequent reported symptom was abdominal pain (91.7%). Of the 32 patients with a previous history of treatment for CE, 27 (84.4%) were treated surgically and 5 (15.6%) received medical treatment with albendazole. Nobody reported previous percutaneous treatment. Of these previously treated CE patients, 11 (34.4%) had active cysts on US examination, but unfortunately it was not possible to assess whether these were new infections or relapses after treatment, due to the lack of medical documentation. The prevalence and distribution of CE lesions by age group and gender is shown in Fig 3A.

The 94 patients with abdominal CE cysts had a total of 131 CE cysts (mean 1.4 CE cysts per patient; range 1–8). Of these, 84 (89.4%) patients had CE cysts only in the liver; 3 (3.2%) in the liver and another localization, notably the peritoneum in 2 patients and the spleen in 1 patient; and 7 patients (7.4%) had CE cysts only in extra-hepatic locations, notably the peritoneum in 3 patients, the spleen in 3 patients, and the kidney in 1 patient. The distribution of hepatic CE cysts by stage is shown in Fig 3B. Liver CE cysts were most frequently inactive (55.8% were CE4-CE5 stages), followed by stages CE3b (19.2%), CE1 (13.3%), CE2 (8.3%) and CE3a (3.3%). As CE has a slow progressive evolution and the exposure may vary due to gender and age-related activities, we investigated the distribution of cyst stages according to these variables. As it was not possible to discriminate between relapse and reinfection in previously treated patients, the distribution of CE stages by sex and age was analysed here only in the 70 previously untreated patients (68 newly diagnosed and 2 known infected but never treated patients). We found that the relative frequency of inactive cysts increased with age, while that of active cysts decreased with age. The risk of having an active cyst significantly decreased with age (p = 0.003 OR 0.33 CI 0.16–0.69). Results are shown in Fig 3C and 3D. Of the 29 patients who had been previously treated for abdominal CE, 17 (58.6%) had only residual cavities/scars or inactive CE4-CE5 cysts on US examination (7 [24.1%] only residual cavities/scars; 10 [34.5%] inactive cysts), while 12 (41.4%) had cysts in active stage. However, as stated above, it was not possible to discriminate between relapse and reinfection.

### Serology and PCR

**Fig 4:**
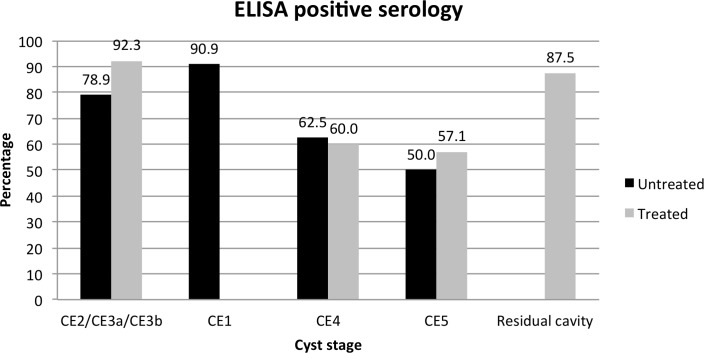
Percentage of untreated and previously treated CE patients with ELISA positive serology according to cyst stage.

Sera from patients with CE were tested by ELISA and WB and the results analysed by cyst stage group as detailed in the methods. Of the 32 patients previously treated for CE, 78.2% had positive serology on both ELISA and WB. Of note, among these previously treated patients, only 1 of the 8 patients having just residual cavities/scars from previous surgery for CE was seronegative, and one seronegative patient had a CE3b cyst in the spleen. Of the 70 previously untreated patients, 21 (30%) were seronegative on both ELISA and WB, 6 (8.6%) had only positive ELISA serology, and 41 (58.6%) were seropositive with both tests. For 2 patients data regarding serology were not obtained. The percentage of ELISA positive results according to cyst stage in untreated and previously treated patients is shown in Fig 4. Of note, only 1 patient was receiving albendazole treatment at the time of blood sampling. One (4.3%) of people that were excluded from having CE had ELISA and WB positive results.

Cyst fluid was obtained from 13 lesions and evaluated by microscopy and PCR. Three suspect lesions were excluded from being CE upon the result of negative microscopy and PCR; of note, one of these patients had a suspect liquid cyst together with a CE5 cyst and positive serology, therefore the non-parasitic nature of the liquid cyst could have not been determined by the serology result only. The negativity of microscopy and PCR of aspirated liquid from 4 lesions of patients with a previous history of surgery and images suspect for relapse allowed to exclude relapse and classify the lesions as residual cavities. All the remaining 6 cyst fluids had protoscoleces on microscopy. Of these, 5 were also positive by PCR and identified as genotype G1 (*E*. *granulosus* sensu stricto; sheep strain).

## Discussion

CE is endemic in Mediterranean countries, including Morocco, however its real prevalence, incidence and burden are difficult to estimate. This is due to the uneven distribution of transmission areas in endemic countries, the high proportion of asymptomatic infected individuals and symptomatic patients living in resource-poor areas with logistical and/or economic constraints, who never reach medical attention, and the underreporting of diagnosed cases. Furthermore, commonly used measures such as surgical case incidence are not appropriate to evaluate the dynamics of a chronic and clinically complex infection such as CE. A comprehensive evaluation of infection and disease burden, and of transmission risk factors, is at the basis of the decision, by public health authorities, upon the implementation of control programmes and rationalization of diagnosis and treatment recommendations. Population US surveys using portable and relatively inexpensive scanners allow obtaining more comprehensive, accurate and detailed information on infection prevalence and stage distribution of CE. The finding of early active cyst stages arguably reflects transmission pressure. Furthermore, US is non-invasive and repeatable, thus it can be used to monitor the effectiveness of control interventions [17]. Training of local physicians on focused assessment with US of CE and the rational allocation of infected patients to clinical management options allow for the reduction of costs associated with the need of travel to tertiary care facilities and provide an efficacious and less expensive tool for patients and health care systems [13]. Finally, community-based US surveys may constitute a useful educational activity, raising awareness of the importance of the infection in the population living in endemic areas [14].

CE is endemic throughout the Mediterranean, which is an area of intense migration, and infected patients may be diagnosed long after infection and in a different country from where it was acquired. Recent data from the WHO Collaborating Centre on Clinical Management of CE in Pavia, Italy, showed that 38.2% of the 203 patients with CE followed by the Centre between January 2012 and February 2014 were foreign-born, and Morocco was the country of birth of the majority (27.2%) of these patients [18]. In this work, carried out through an Italian-Moroccan partnership, we estimated the prevalence and the characteristics of human abdominal CE by means of a community-based US screening in the provinces of Ifrane and El Hajeb, Meknes-Tafilalet region, Mid Atlas, an area among the most endemic in Morocco for both human and animal infection. In four 2-day campaigns using 8 portable US machines operated by experienced clinicians, we screened 13.1% of the population of the two investigated rural communes of Timahdit and Ain Louh in Ifrane and 17.3% of the two investigated rural communes of Bouderbala and Sebt Jahjouh in El Hajeb. This study had several limitations. First, this sample population included a lower percentage of young (<20 years of age) people and a higher percentage of middle-aged (40–60 years) people compared to the general registered population of the investigated provinces; also, more females than males volunteered to participate to the survey. Second, the screened sample was self-selected due to the voluntary nature of the participation, which could have biased the estimate in either direction. A random sampling was not considered suitable here due to concerns about the acceptability of such sampling method by the population. Third, children younger than 10 years of age were excluded from the target population due to practical constraints regarding treatment of CE in this age range population.

We found an overall CE prevalence of 1.9% (CI 1.6%-2.4%), a figure almost double compared to what reported by Macpherson and colleagues [6], who carried out a population US survey in the provinces of Ifrane and Khenifra, Mid Atlas, in 2000–2001, observing a prevalence of 1.1% (CI 0.9%-1.3%). This discrepancy could be due to a real increase in the infection pressure in the absence of control measures, or derive from differences in the investigated areas and target populations. Indeed, CE infection burden is difficult to estimate also due to the uneven distribution of transmission even within relatively small areas. Although a direct comparison of the two surveys is therefore not possible, these results demonstrate, as expected, an on-going transmission of the infection in the region. In our survey, CE prevalence in Ifrane was twice that in El Hajeb, with no differences between rural communes within provinces. The analysis of the prevalence of infection by age and gender showed the highest values in males aged 21–40 years, with comparable values in males and females within age groups. These results are in line with what found by Macpherson and colleagues [6]. However, we observed a decrease in infection prevalence in the population aged > 40 years. This was not explained by an increase in treatment rate with age, as prevalence was calculated including both subjects untreated and previously treated for CE. Possible explanations may be related to the structure of the resident population (e.g. more people aged 40–60 years may be emigrated from rural areas) and/or to the spontaneous resolution of the infection with time, with disappearance of the lesions possibly combined with the acquisition over time of some protective immunity to new infections [19].

When we investigated risk factors using multivariable analysis, we found that only province of residence (Ifrane) and dog ownership were significantly associated with CE infection, while age and gender were not. Of note, Ifrane is the province with more extensive livestock breeding activity and meat production (i.e. slaughter activity). These results are different from previous work, where dog ownership was generally not associated with CE infection in studies investigating adults or communities, while gender and source of water were consistently associated with CE infection [20–24]. These results, however, are consistent with those reported in the recent systematic review and meta-analysis of potential risk factors associated with CE infection [1]. In the investigated area, “dog ownership” may be not intended in the same strict manner as in Europe. Indeed, from the analysis of the answers to the questionnaire, we noticed that at-risk practices such as home slaughter, unsafe disposal of livestock viscera in public places (e.g. garbage or open fields accessible to dogs), and feeding of dogs with raw viscera, were carried out independently of the strict ownership of livestock and dogs. Possibly, a more precise investigation of the type of contact with dogs rather than the general terminology of “dog ownership” could be more informative on the role of direct contact with dogs in the transmission of CE. Our result that province of residence was the only risk factor consistently associated with CE infection suggest that environmental contamination is likely the main factor responsible for CE transmission in this area. A more detailed assessment of habits and analysis of materials (water, soil, food) would be necessary to try individuating the actual route of infection, as also suggested by Possenti et al [1]. However, the acquisition of infection a long time before diagnosis makes the evaluation of such causality very difficult.

When considering CE infection in previously untreated patients, we observed that CE cysts were predominantly in an inactive stage, supporting the findings of previous longitudinal and observational studies showing that cysts evolve spontaneously to inactivation over time [25–30]. However, active cyst stages, including CE1 cysts (i.e. cysts likely acquired in recent times), were present also in adult age groups, as also found by Macpherson and colleagues in the same area [6], indicating acquisition of infection even in adulthood, although at lower rate. This may be due to the acquisition of some degree of immunity or to a decrease in exposure to infection at older age. In any case, these results are of particular importance. First, the predominance of inactive cysts in the sample population and the increase of the presence of inactive cysts with increasing age support the need for a stage-specific approach. Indeed, most cysts evidently evolve spontaneously to inactivation. As a consequence, aggressive invasive therapy, when not needed because of symptoms or complications, is not appropriate in most cases, and spontaneously inactivated cysts (the majority of those found in our population) do not have to be treated at all [8, 9]. Second, the evidence of acquisition of infection even in adulthood implies that a benefit from control programmes may be observed in all age groups, as already observed by Beard [31]. Indeed, the assumption that most cysts are acquired at early age but only evident after years hindered control measures, as this implied that investments for decades were needed before visible results may be obtained [31]. On the contrary, the practical consequence of the finding that adults are also susceptible and that latency between infection and diagnosis may be shorter than believed, is that expenditures for control may be encouraged by the expectation of early measurable benefits to the whole community.

To conclude, our results show a high prevalence and on-going transmission of CE in the investigated provinces of Ifrane and El Hajeb, Mid Atlas of Morocco. A plan for a control program in Morocco was envisaged after the study on the incidence of hospitalized CE cases at the national level (1980–1992) carried out by the Ministry of Health. In 2003 a national register of hospital CE cases was implemented. In 2004, an inter-ministerial committee for the control of CE was established in Morocco. The activities envisaged in the control program included health education, improvement of general hygiene, strengthening of the case registration system, control of the stray dog population, and screening and early treatment of patients. The intersectoral strategy was developed in 2007 and the activities started in 2009, but so far only improvement of human CE case report system has been implemented. Our data confirm the need for control activities in the area by national health authorities, through the full implementation of envisaged activities, and possibly the inclusion of other measures [32] after revision of the current plan, in the light of the difficulties and constraints to the implementation of such control programs. Also, our results encourage the acceptance and use of diagnostic-based algorithms using imaging rather than serology, and a stage-specific management approach for CE. Indeed, with presently available tools cyst activity cannot be assessed by serology, as more than 50% of subjects with spontaneously inactivated cysts (which remain stably inactive in >97% of cases [9]) and more than 85% of subjects with post-surgical residual cavities/scars had a positive serology in our population. These patients should be correctly diagnosed as not having an active infection, and should be solely monitored over time, avoiding expensive and risky treatments such as surgery [8, 9]. Focused training of local physicians on US in CE diagnosis, management options, and follow-up would be a valuable tool. Indeed, in Morocco, surgery is still almost the sole treatment option offered to patients [5], as also shown by our results of treatment type reported by previously treated patients, and compliance to current recommendations on the clinical management of CE is still very low [10, 12]. Our experience also shows that population-based screening campaigns are useful to assess the prevalence, dynamics, and risk factors of CE in endemic areas, and may provide the favourable occasion to implement focused training for local health care workers and health education for general population. In this study, children younger than 10 years of age were excluded from the target population due to practical constraints regarding treatment of CE in this age range population. However, the inclusion of a younger population would be important in studies preliminary to the implementation of control activities, to provide baseline data, and for the monitoring of infection incidence once the program is in ongoing.

A highly coordinated multidisciplinary team is pivotal and a systematic pre-survey planning is absolutely required to address research questions and implement operational activities on an infection such as CE whose management is the exemplification of the "One Health" approach. However, the building and coordination of such teams is difficult, and requires time and continuous work over time with the same group. Also, the individuation and agreement on the relative share of costs and coordination between human and veterinary health services is an issue [33]. In this regard, it would be desirable to include all or part of the same staff in future surveys, to take advantage of the experience gained and lessons learned from previous activities through the identification of critical points after completion of field work (e.g. here the administration of the education session before the questionnaire for logistical convenience posed then problems in the interpretation of answers to some questions of the questionnaire). A pilot testing of the risk factors questionnaire and the inclusion of a social scientist in the team are also advisable to better adapt epidemiological tools to the habits and structure of the target communities, improve attendance from all age groups and genders and advise on the social acceptability of different sampling methods [34].

## Supporting information

S1 Checklist STROBE checklist. (DOCX)

S1 Dataset Original data file. (XLS)
